# Multimerization is required for antigen binding activity of an engineered IgM/IgG chimeric antibody recognizing a skin-related antigen

**DOI:** 10.1038/s41598-017-08294-2

**Published:** 2017-08-15

**Authors:** Kwesi Teye, Koji Hashimoto, Sanae Numata, Kunihiro Ohta, Marek Haftek, Takashi Hashimoto

**Affiliations:** 1 Kurume University Institute of Cutaneous Cell Biology, Kurume, Fukuoka Japan; 2 Department of Life Sciences, Graduate School of Arts and Sciences, The University of Tokyo, Tokyo, Japan; 3 Division of Innovation and Education, Iwate Tohoku Medical Megabank Organization, Disaster Reconstruction Center, Iwate Medical University, Iwate, Japan; 4 University of Lyon 1, EA 4169 and CNRS, Lyon, France

## Abstract

Monoclonal antibodies offer great tools for research. We encountered a potentially useful mouse IgM monoclonal antibody whose antigen is expressed in normal skin but lost in human skin cancer. Because IgM is difficult to work with and the antigen was unknown, we decided to convert the IgM (µ) to IgG (γ) version. After cDNA for the antibody was obtained by RACE PCR, we made a series of molecules with different combinations of IgM and IgG domains. Whereas V_H_-Cµ1-Cµ2-Cγ3 and V_H_-Cµ1-Cµ2-Hinge-Cγ2-Cγ3 functionally bound to the antigen, V_H_-Cγ1-Hinge-Cγ2-Cγ3, V_H_-Cµ1-Hinge-Cγ2-Cγ3, and V_H_-Cµ1-Cµ2-Cγ2-Cγ3 did not. Gel filtration analyses revealed that the functional molecules tend to form multimers and the multimeric forms retained antigen binding activity. Furthermore, the mutation of amino acid residue p.309Q > C of mouse IgG and addition of IgM tailpiece to the C-terminus of the molecules induced multimer formation, dramatically enhanced antibody functionality and all non-functional molecules became strongly functional. The functional molecules could be bound by protein A/protein G and other IgG specific reagents and therefore should be useful for further characterization of the antigen. Our study revealed that multimerization of converted IgM is functionally important for antigen binding activity of engineered IgM/IgG chimeric antibodies.

## Introduction

IgG by far is the most common Ig used in research and many reagents are available for use with IgG. IgM, being a very large molecule, is difficult to work with in terms of reagent availability, purification and specificity. IgM does not bind to common bacterial protein A and protein G^1^, which are often used in co-immunoprecipitation applications. Although IgM binds to bacterial protein L^2, 3^, the binding occurs through the light chain^2, 3^ and, in our hands, it is inefficient for immunoprecipitation, particularly when the antigen is large and there is a probability of steric hindrance. Therefore, when an antibody is of IgM type and RNA from its hybridoma is available, it is often desirable to convert it into an IgG format by cloning its cDNA to explore its full potential for many purposes.

An IgM monoclonal antibody, which is named KM48 and recognizes a skin related antigen, was generated after immunization of mouse with normal human epidermal cell suspension^4^. Immunocytochemical studies revealed that the antibody recognized an antigen associated with human keratinocyte plasma membrane and, in particular, with desmosomes^4, 5^, which are cell adhering apparatus that maintain normal skin integrity^6^. Studies also showed that the antigen is expressed in normal human skin but defective in human squamous cell carcinoma (SCC)^7^, suggesting that the antibody recognizes a potentially important antigen in the skin. Biochemical analyses revealed that the antigen is different from known desmosomal proteins^8^ and therefore may react with an unidentified novel molecule.

However, attempts to identify the antigen for KM48 have failed, and the failure was mainly attributed to the antibody being of IgM class, which has limited usability for applications, such as immunoprecipitation. To overcome the obstacle of limited usability of the IgM antibody, we decided to convert the IgM antibody into an IgG format to enable us to identify or confirm its antigen using immunoprecipitation. Conversion of one form of Ig to another is usually done by joining the variable regions of the antibody of interest to the constant regions of a desired isotype^9–11^. However, conversion of IgM to IgG is problematic in some situations^11, 12^.

In this study, we constructed and produced functional IgM/IgG hybrid molecules of KM48 by combining different portions of the µ chain and different portions of the γ chain. Eventually, we identified Ig domains and structures that are functionally required for antigen binding in engineered IgM/IgG antibodies.

## Results

### RACE PCR of IgM heavy and light chains and PCR of mouse IgG2a Cγ1 to Cγ3

**Figure 1:**
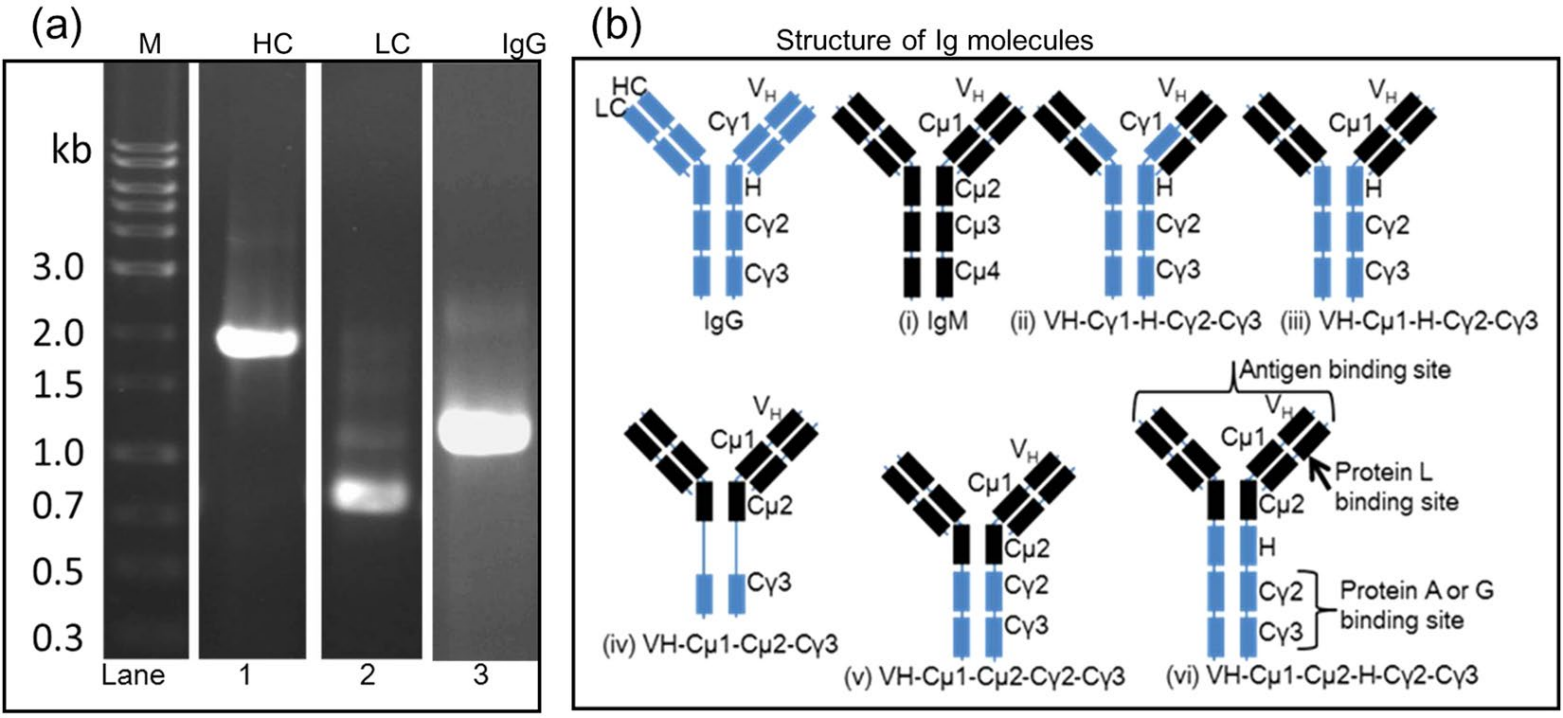
Cloning of IgM cDNA and construction of various IgM/IgG chimeric molecules. (**a**) RACE PCR amplification of heavy (HC) and light (LC) chains of mouse IgM from hybridoma cDNA and amplification of IgG2a (IgG) from normal mouse spleen cDNA. 1-kb ladder (M) was used as molecular weight marker. (**b**) Schematic structures of IgG, IgM and chimeric IgM/IgG molecules analyzed in this study. The domains are not drawn to scale. Antigen, protein A, protein G and protein L binding sites are indicated. Each molecule was given a serial number of (i), (ii), (iii), (iv), (v) and (vi).

By rapid amplification of cDNA ends (RACE) PCR, approximately 2 kb product was obtained for IgM heavy chain and a 0.7 kb product was obtained for the light chain from our hybidoma RACE cDNA pool (Fig. 1a, lanes 1 and 2). Sequencing of the products confirmed the heavy chain product to be IgM and the light chain to be kappa cDNA (data not shown). However, the hybridoma cells also produced a previously reported non-functional kappa light chain^13^, which was excluded from further analysis. PCR was also used to successfully amplify the constant region of mouse IgG2a from normal mouse spleen cDNA (Fig. 1a, lane 3). The predicted size was approximately 1 kb.

### Production and characterization of various IgM/IgG hybrid molecules

**Figure 2:**
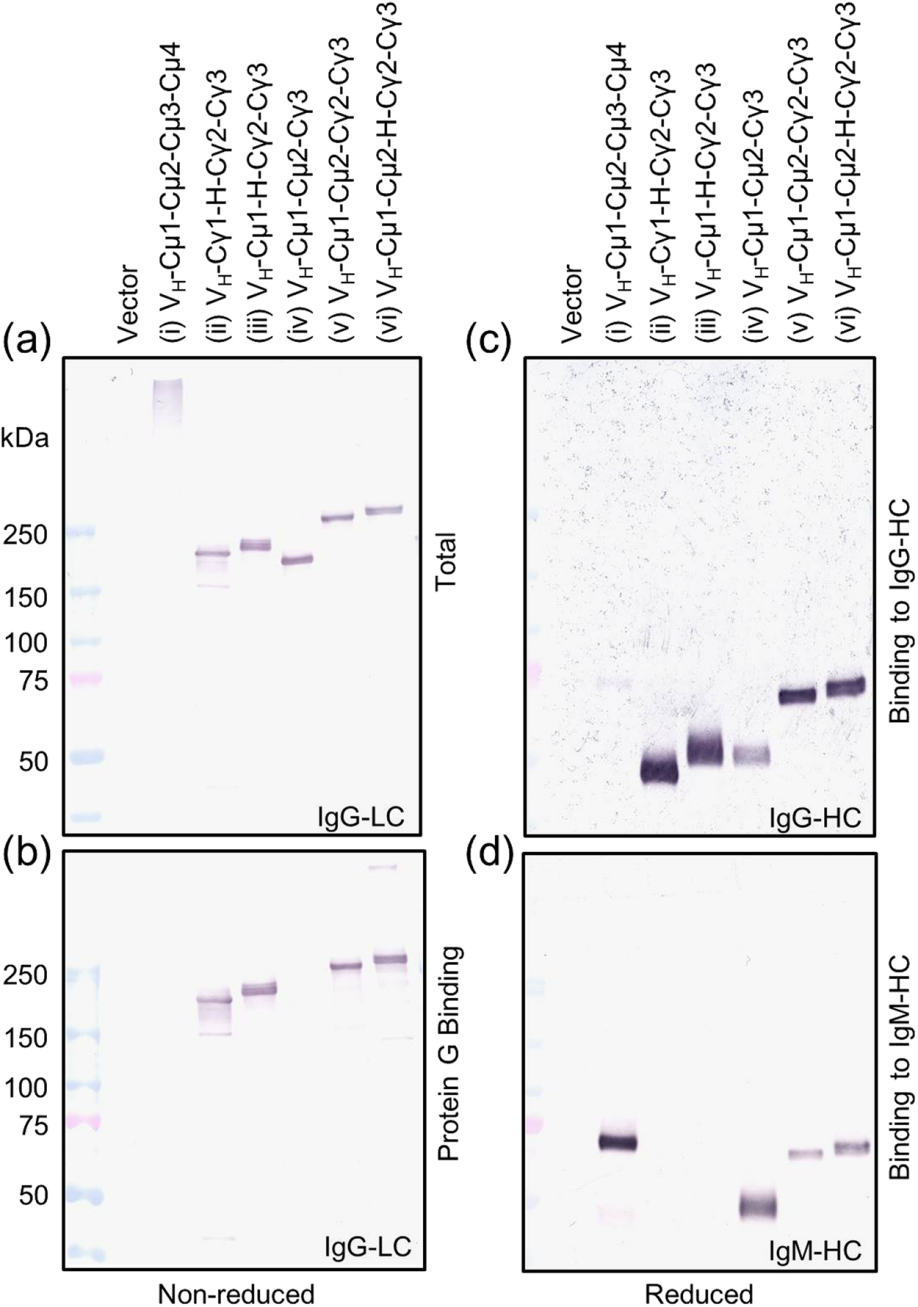
Production and characterization of various IgM/IgG hybrid molecules. (**a**) Non-reducing Western blotting analysis of culture supernatant of HEK293T transfected with cDNA for the indicated IgM/IgG molecules using anti-mouse kappa chain specific-HRP. (**b**) Analysis of Protein G binding to various IgM/IgG hybrid molecules. Western blotting was performed under non-reducing condition using anti-mouse kappa chain specific-HRP. (**c** and **d)** Analysis of binding of anti-mouse IgG (**c**) and anti-mouse IgM (**d**) to the heavy chains of various IgM/IgG hybrid molecules. ALP-conjugated anti-mouse IgG or IgM was used as secondary antibody. Cropped blots are shown and full length blots are shown in Supplementary Fig. S1.

Traditionally, antibody conversion protocols call for joining the variable region of antibody of interest to the constant region of the desired Ig type^9, 14^. Due to the perceived problems with conversion of IgM to IgG, we constructed and analyzed several molecules (Fig. 1b). For ease of identification, each molecule was given a serial number from (i) to (vi) (Fig. 1b). The V_H_ domain and κ light chain were common to all molecules but the constant region of the heavy chain was made up of different portions of IgM and different portions of IgG2a (Fig. 1b). Western blotting analysis of cell culture supernatant of transiently transfected HEK293T cells indicated that all recombinant antibodies were successfully expressed and assembled in the cells (Fig. 2a). Apart from (i) V_H_-Cµ1-Cµ2-Cµ3-Cµ4, which was very large, all other molecules resolved between 150 and 250 kDa, with occasional high molecular weight products in some cases (Fig. 2a,b). Whereas (i) V_H_-Cµ1-Cµ2-Cµ3-Cµ4 and (iv) V_H_-Cµ1-Cµ2-Cγ3 did not bind to protein G, all other molecules bound to protein G, suggesting that Cγ2 and Cγ3 domains of IgG are required for protein G binding (Fig. 2b and Table 1). Anti-mouse IgG bound moderately to the heavy chain of (iv) V_H_-Cµ1-Cµ2-Cγ3 but not (i) V_H_-Cµ1-Cµ2-Cµ3-Cµ4 (Fig. 2c). On the other hand, anti-mouse IgG bound well to the heavy chain of the other molecules containing the Cγ2 and Cγ3 domains of IgG2a (Fig. 2c and Table 1). Anti-mouse IgM bound well to (i) V_H_-Cµ1-Cµ2-Cµ3-Cµ4 and (iv) V_H_-Cµ1-Cµ2-Cγ3, moderately to (v) V_H_-Cµ1-Cµ2-Cγ2-Cγ3 and (vi) V_H_-Cµ1-Cµ2-Hinge-Cγ2-Cγ3, but did not bind to (ii) V_H_-Cγ1-Hinge-Cγ2-Cγ3 and (iii) V_H_-Cµ1-Hinge-Cγ2-Cγ3 (Fig. 2d and Table 1). These results suggested that IgM/IgG hybrid molecules with properties of IgG were successfully constructed and could be stably expressed in the HEK293T cells.

**Table 1:** Summary of properties of recombinant IgM and IgM/IgG molecules.

	(i) V_H_ -Cµ1 -Cµ2 -Cµ3 -Cµ4	(ii) V_H_ -Cγ1 -Hinge -Cγ2 -Cγ3	(iii) V_H_ -Cµ1 -Hinge -Cγ2 -Cγ3	(iv) V_H_ -Cµ1 -Cµ2 -Cγ3	(v) V_H_ -Cµ1 -Cµ2 -Cγ2 -Cγ3	(vi) V_H_ -Cµ1 -Cµ2 -Hinge -Cγ2 -Cγ3
Protein G binding	**−**	**+**	**+**	**−**	**+**	**+**
IgG-HC binding	**−**	**+**	**+**	**+**	**+**	**+**
IgM-HC binding	**+**	**−**	**−**	**+**	**+**	**+**
Skin IF	+	−(+)*	−(+)*	+	−(+)*	+(+)*
EE WB	**+**	**−**	**−**	**+**	**−**	**+**

HC, heavy chain, IF, Immunofluorescence, EE, epidermal extract, WB, Western blotting, H, Hinge, *(With single or double 309 C and µTP mutations).

### Some IgM/IgG hybrid molecules have function

**Figure 3:**
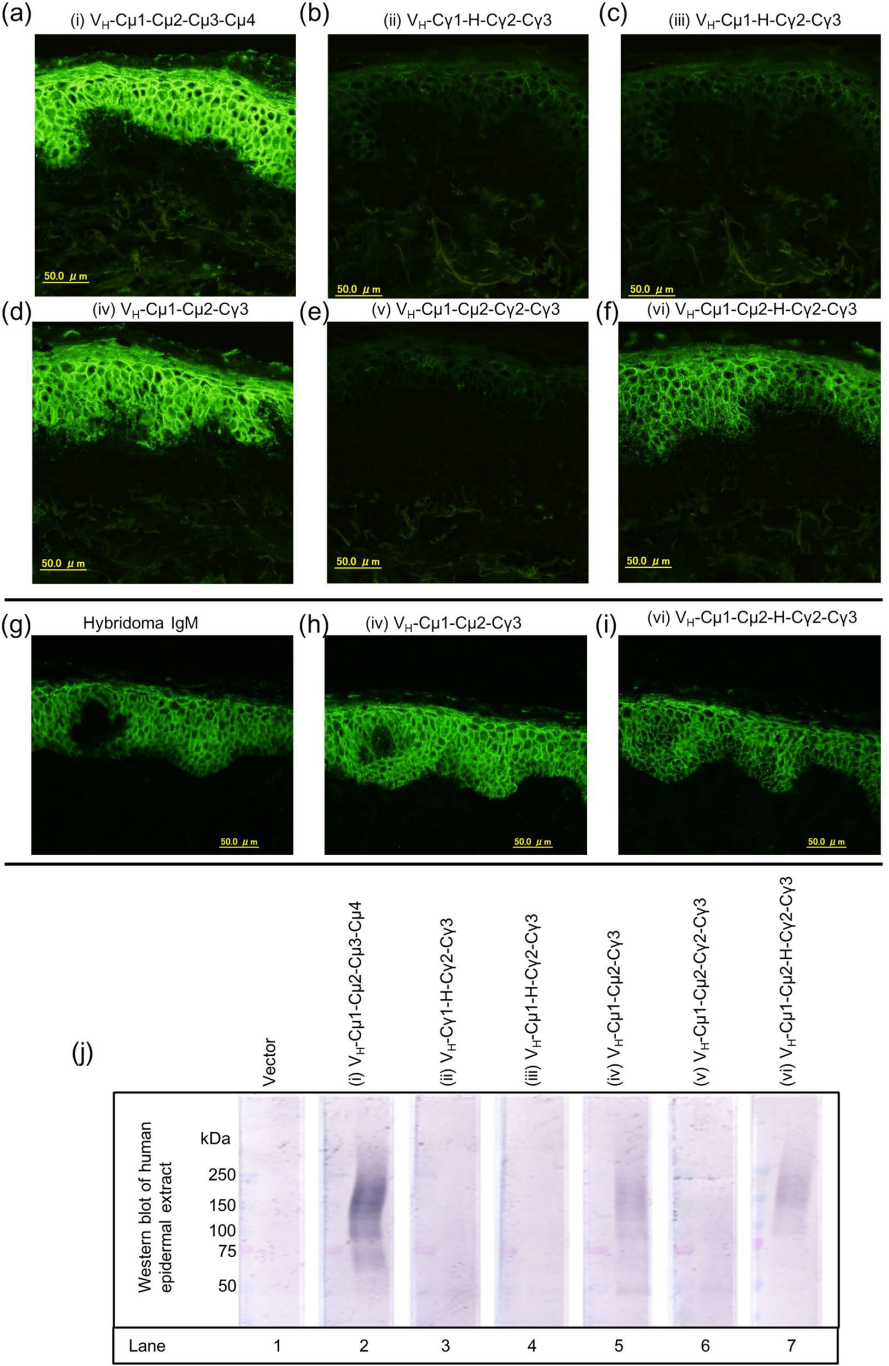
Immunofluorescence and Western blotting analysis of normal human skin proteins using various IgM/IgG hybrid molecules. (**a**–**f**) Immunofluorescence of normal human skin using the indicated IgM/IgG hybrid molecules as primary antibodies. (**g**–**i**) Comparison of antigen binding activity between the original hybridoma-derived IgM molecule and the two functional IgM/IgG molecules. (**j**) Western blotting analysis of normal human epidermal extract using the indicated IgM/IgG hybrid molecules as primary antibodies. The antibody detects a range of glycosylated epidermal antigen ranging from 75–250 kDa. Cropped blots are shown and full length blots are shown in Supplementary Fig. S2.

To determine whether or not the constructed molecules were functional, we performed immunofluorescence of normal human skin and the results showed that some of the antibodies were functional (Fig. 3a–f). The analysis showed that the full-length IgM molecule, designated as (i) V_H_-Cµ1-Cµ2-Cµ3-Cµ4, functionally bound to the epidermis of normal human skin (Fig. 3a), suggesting the correct IgM molecule was successfully cloned from the hybridoma cells. (iv) V_H_-Cµ1-Cµ2-Cγ3 (Fig. 3d) and (vi) V_H_-Cµ1- Cµ2-Hinge-Cγ2-Cγ3 (Fig. 3f) were also functional. We noted that the binding of (vi) V_H_-Cµ1-Cµ2-Hinge-Cγ2-Cγ3 was slightly weaker than those of (i) V_H_-Cµ1-Cµ2-Cµ3-Cµ4 and (iv) V_H_-Cµ1-Cµ2-Cγ3 (Fig. 3a,d,f). On the other hand, (ii) V_H_-Cγ1-Hinge-Cγ2-Cγ3 (Fig. 3b), (iii) V_H_-Cµ1-Hinge-Cγ2-Cγ3 (Fig. 3c) and (v) V_H_-Cµ1-Cµ2-Cγ2-Cγ3 (Fig. 3e) all failed to bind to the epidermis-related antigen.

We also compared the binding activities of the functional molecules with that of the original hybridoma-derived IgM molecule. Immunofluorescence of normal human skin revealed that the reactivities of the recombinant IgM/IgG molecules were similar to that of the original IgM molecule (Fig. 3g–i).

We also performed Western blotting of normal human epidermal extract to examine the reactivities of the antibodies. Similar to the results of immunofluorescence, the Western blot analysis showed that (i) V_H_-Cµ1-Cµ2-Cµ3-Cµ4, (iv) V_H_-Cµ1-Cµ2-Cγ3 and (vi) V_H_-Cµ1-Cµ1-Hinge-Cγ2-Cγ3 showed positive reactivity, while (ii) V_H_-Cγ1-Hinge-Cγ2-Cγ3, (iii) V_H_-Cµ1-Hinge-Cγ2-Cγ3 and (v) V_H_-Cµ1-Cµ2-Cγ2-Cγ3 were negative (Fig. 3j). The combined results of immunofluorescence and Western blotting suggested that (i) V_H_-Cµ1-Cµ2-Cµ3-Cµ4, (iv) V_H_-Cµ1-Cµ2-Cγ3 and (vi) V_H_-Cµ1-Cµ2-Hinge-Cγ2-Cγ3 are functional but (ii) V_H_-Cγ1-Hinge-Cγ2-Cγ3, (iii) V_H_-Cµ1-Hinge-Cγ2-Cγ3 and (v) V_H_-Cµ1-Cµ2-Cγ2-Cγ3 are not functional.

### Functional IgM/IgG molecules form multimers

**Figure 4:**
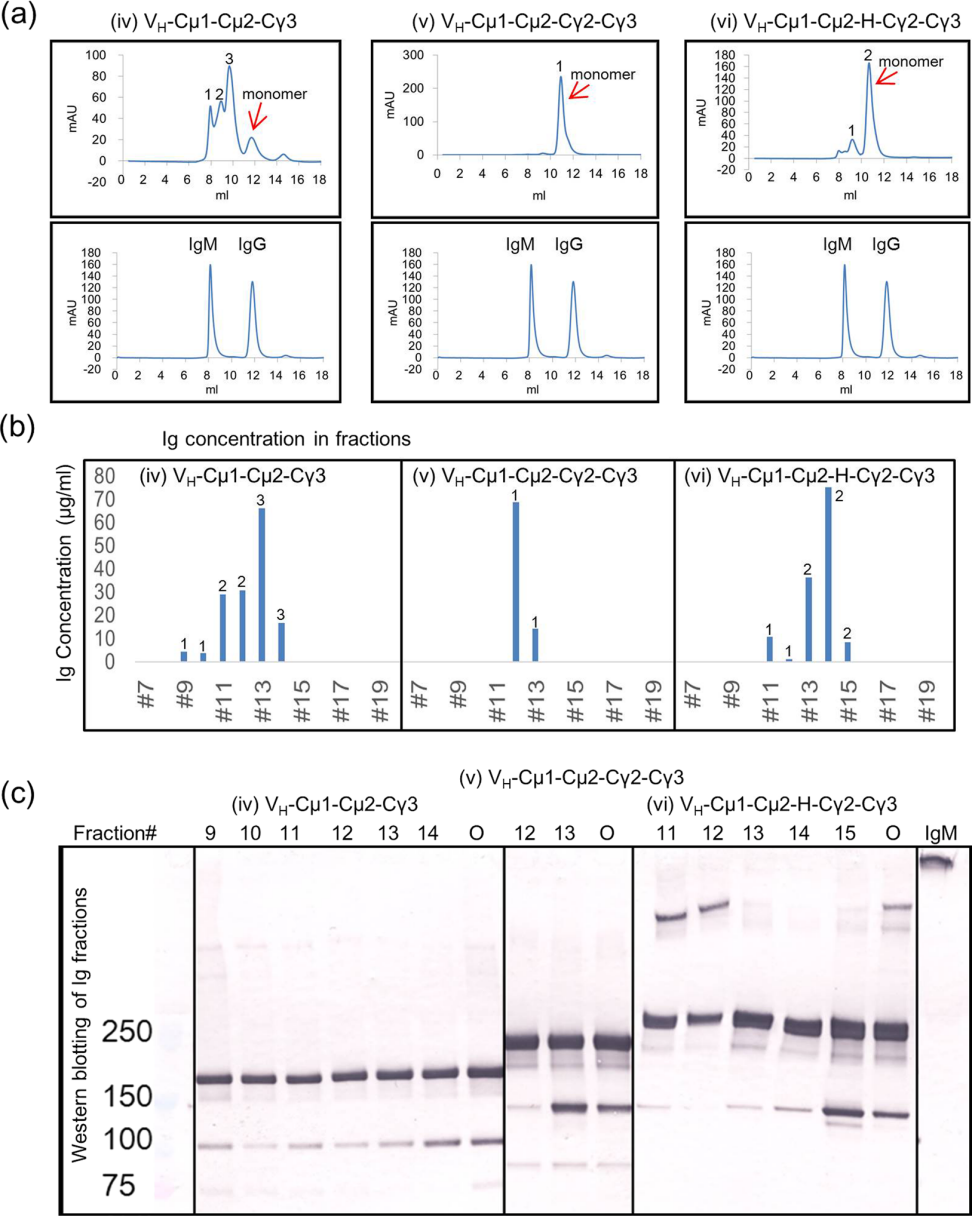
Results of gel filtration, ELISA and Western blotting analyses of IgM/IgG molecules. (**a**) Presentation of gel filtration chromatographs for the indicated IgM/IgG hybrid molecules. As a standard, the mixture of recombinant mouse IgG2a (IgG) and original KM48 IgM antibody (IgM) was also analyzed. For ease of comparison, the chromatograph for IgM/IgG mixture was aligned under each of the chromatographs for the other molecules. The elution positions of monomers are indicated. (**b**) The concentrations of Ig in gel filtration fractions. Concentrations were determined by our novel IgM/IgG sandwich ELISA system for the indicated IgM/IgG molecules. Fractions 7 to 20 were analyzed by ELISA. (**c**) Non-reducing Western blotting analysis of Ig in fractions. Fractions with sufficient Ig concentration were used. Each non-fractionated sample (O) and hybridoma-derived IgM molecule (IgM) were used as controls. Cropped blots are shown and full length blots are shown in Supplementary Fig. S3.

Since IgM normally polymerizes as pentamer with J-chain or hexamer without J-chain^15^, we performed gel filtration to examine the multimeric state of the recombinant antibodies. We chose the 3 molecules containing the Cµ2 domain i.e. (iv) V_H_-Cµ1-Cµ2-Cγ3, (v) V_H_-Cµ1-Cµ2-Cγ2-Cγ3 and (vi) V_H_-Cµ1-Cµ2-Hinge-Cγ2-Cγ3 because both functional IgM/IgG chimeric antibodies, (iv) V_H_-Cµ1-Cµ2-Cγ3 and (vi) V_H_-Cµ1-Cµ2-Hinge-Cγ2-Cγ3 have the Cµ2 domain. By gel filtration analysis, we found that the functional molecules, but not the non-functional molecule, tend to form multimers (Fig. 4a). The sizes of the multimers were between those of IgM and IgG (Fig. 4a). Multimer formation was much profound in (iv) V_H_-Cµ1-Cµ2-Cγ3 than in (vi) V_H_-Cµ1-Cµ2-Hinge-Cγ2-Cγ3. The amount of monomer was very low in (iv) V_H_-Cµ1-Cµ2-Cγ3 (Fig. 4a). The monomer was predominant in (vi) V_H_-Cµ1-Cµ2-Hinge-Cγ2-Cγ3 and multimers were virtually absent in (v) V_H_-Cµ1-Cµ2-Cγ2-Cγ3 (Fig. 4a). Because the samples were fractionated manually, the volume of each fraction was not constant. Therefore, the concentrations of Ig in the fractions of the peaks were measured by our novel sandwich ELISA (Fig. 4b). The results confirmed that Ig was present in different fractions with different sizes in (iv) V_H_-Cµ1-Cµ2-Cγ3, in (vi) V_H_-Cµ1-Cµ2-Hinge-Cγ2-Cγ3 to a lesser extent and very few fractions in (v) V_H_-Cµ1-Cµ2-Cγ2-Cγ3 (Fig. 4b). Western blotting analyses of fractions with sufficient Ig concentrations confirmed that larger molecules were enriched in earlier fractions in (iv) V_H_-Cµ1-Cµ2-Cγ3 and (vi) V_H_-Cµ1-Cµ2-Hinge-Cγ2-Cγ3 but not in (v) V_H_-Cµ1-Cµ2-Cγ2-Cγ3 (Fig. 4c). However, significant amounts of monomeric forms were also detected, suggesting that the molecules formed non-covalent associations.

**Figure 5:**
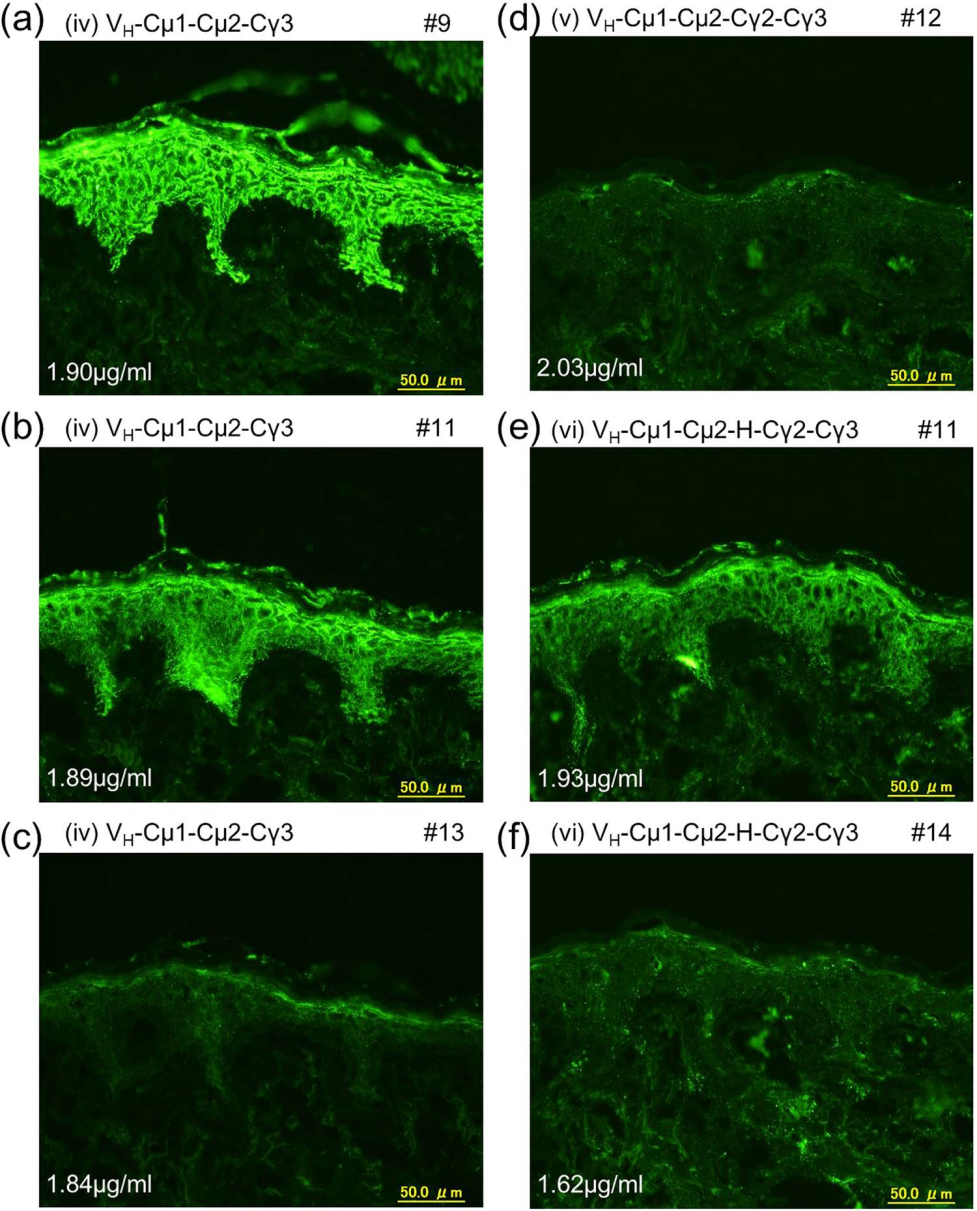
Immunofluorescence of normal human skin. (**a**–**f**) Immunofluorescence was performed with gel filtration fractions of the indicated IgM/IgG molecules as primary antibodies. The indicated final Ig concentration was determined by ELISA.

By immunofluorescence of normal human skin using representative fractions obtained from gel filtration analysis, we found that Ig multimers, rather than the monomers, appeared to retain the antigen binding activity for both (iv) V_H_-Cµ1-Cµ2-Cγ3 and (vi) V_H_-Cµ1-Cµ2-Hinge-Cγ2-Cγ3 (Fig. 5a–f). None of the fractions of (v) V_H_-Cµ1-Cµ2-Cγ2-Cγ3 stained the epidermis of normal human skin (Fig. 5d). We confirmed that the concentrations of antibody in the reaction mixtures were similar in all cases (Fig. 5a–f), suggesting that the negative reactions were not due to insufficient antibody amount. These results suggested that multimer formation of the chimeric molecules is an important factor for antigen binding activity of the antibodies.

### Mutation p.309Q > C and addition of IgM tailpiece induce multimerization and functionality

Previous studies indicated that mutation p.309 L > C (309 C) of human IgG and addition of IgM tail piece (µTP), consisting of the last 18 amino acids of IgM to the C-terminus can induce multimerization of human IgG^16, 17^. In mouse, the amino acid residue at this position was 309Q. Because multimeric forms of our molecules showed positive reactions (Figs 4 and 5), we mutated IgM/IgG chimeric antibodies to contain 309 C, µTP or both and examined whether these mutations can induce multimer formation and functionality of mouse IgM/IgG chimeric antibodies.

**Figure 6:**
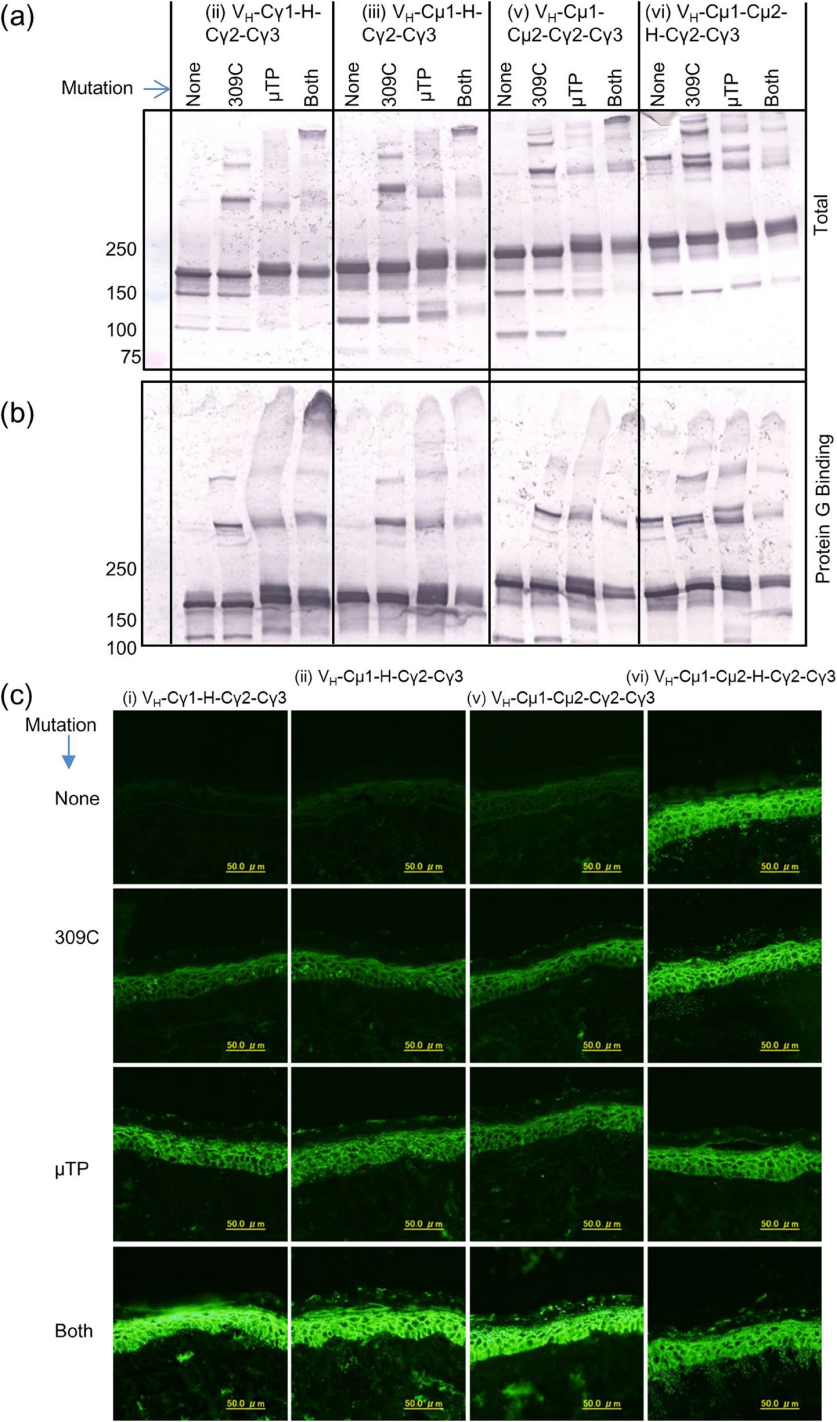
Western blotting, protein G binding and immunofluorescence of human skin using IgM/IgG chimeric molecules with and without 309 C and µTP mutations. (**a**) Non-reducing Western blotting analysis of culture supernatant of HEK293T cells transfected with cDNA for the indicated wild-type and single or double mutant IgM/IgG molecules. (**b**) Analysis of Protein G binding to IgM/IgG hybrid molecules with or without 309 C and µTP mutations. Western blotting was performed under non-reducing condition. (**c**) Immunofluorescence of normal human skin using the indicated wild-type and single or double mutant IgM/IgG chimeric molecules as primary antibodies. Cropped blots are shown and full length blots are shown in Supplementary Fig. S4. In Figs 2 and 6b gels were used. The boundary of the gels is located on the middle vertical line.

Western blot analysis of culture supernatant of transfected HEK293T cells suggested that each and both mutation(s) induced multimer formation of all molecules tested in various patterns (Fig. 6a). Inclusion of both mutations induced formation of a large multimer in 3 out of 4 molecules tested (Fig. 6a). Unexpectedly, multimer formation was less profound in (vi) V_H_-Cµ1-Cµ2-Hinge-Cγ2-Cγ3 with double mutation (Fig. 6a). The multimers also could bind to protein G (Fig. 6b). The large multimer of (ii) V_H_-Cγ1-Hinge-Cγ2-Cγ3 with 309 C and µTP mutations appears to bind to protein G strongly.

**Figure 7:**
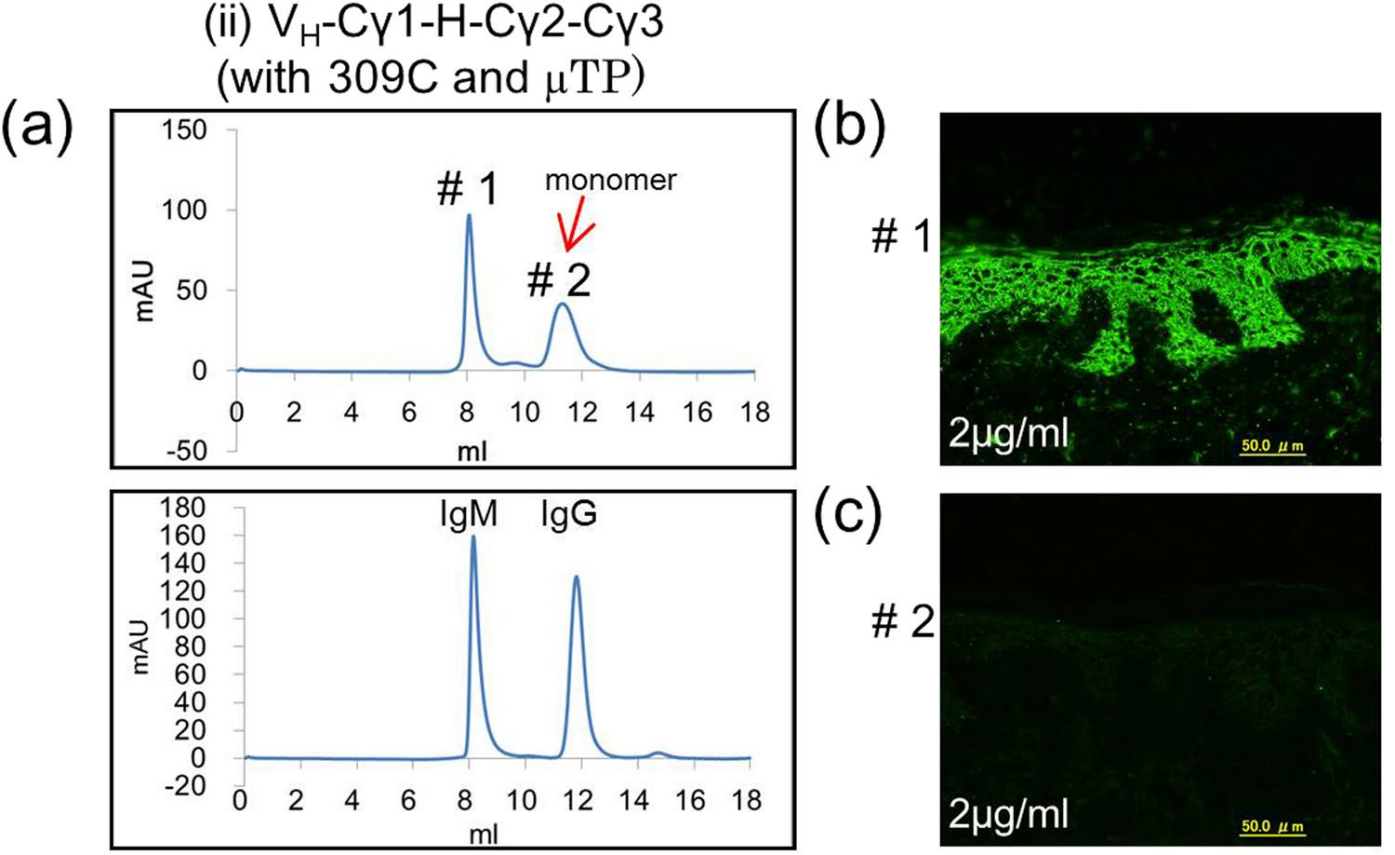
Gel filtration and immunofluorescence of normal human skin using (ii) VH-Cγ1-Hinge-Cγ2-Cγ3 with 309 C and μTP mutations. (**a**) Presentation of gel filtration chromatographs for (ii) VH-Cγ1-Hinge-Cγ2-Cγ3 with 309 C and μTP mutations. Fractions corresponding to the indicated peaks were collected and used for immunofluorescence of normal human skin. The elution position of monomer is indicated. (**b**,**c**) Immunofluorescence of normal human skin using the indicated gel filtration fractions as primary antibodies.

By immunofluorescence analyses of normal human skin, we found that all molecules became functional to varying degrees upon addition of each and both mutation(s) (Fig. 6c). Interestingly and surprisingly, non-mutated non-functional molecules also became functional and signals for their functional molecules became stronger when both mutations were introduced (Fig. 6c and Table 1). We chose the representative molecule of (ii) VH-Cγ1-Hinge-Cγ2-Cγ3 with 309 C and μTP mutations from the viewpoint of configuring all domains with IgG and strong binding to protein G, and performed further analyses. By gel filtration analysis, we found that most of the molecules purified with Protein G form multimers (Fig. 7a). Immunofluorescence analyses using gel filtration fractions revealed that multimers can bind to the antigen (Fig. 7b) whereas the monomers cannot (Fig. 7c). These results clearly demonstrated that multimer formation is required for antigen binding activity of IgM/IgG chimeric antibodies.

## Discussion

In this study, we have successfully converted a mouse IgM monoclonal antibody into functional IgG-like molecules. We demonstrated that multimerization of the molecules is important for antigen binding. Only a few studies have documented the successful conversion of IgM into an IgG format^18, 19^. In most cases, the converted IgM fail to function^11, 12^. Our studies suggest that these failures can be attributed to the lack of the multimeric forms in recombinant IgM/IgG molecules.

Our systematic analysis of several molecules with different IgM/IgG domains provided a clue as to which domains and structures of the molecules are important for antigen binding activity (Figs 1,2 and 3). It is intriguing that (vi) V_H_-Cµ1-Cµ2-Hinge-Cγ2-Cγ3 but not (v) V_H_-Cµ1-Cµ2-Cγ2-Cγ3 was functional. The difference between the 2 molecules is the presence or absence of the 16-amino acid hinge domain of mouse IgG2a. The gel filtration analysis clearly showed that (vi) V_H_-Cµ1-Cµ2-Hinge-Cγ2-Cγ3, but not (v) V_H_-Cµ1-Cµ2-Cγ2-Cγ3, forms multimers (Fig. 4). Previous studies for human IgG2 demonstrated that the cysteine residue(s) in the hinge may be involved in dimer formation^20^. These results suggested that the hinge domain in our IgM/IgG molecule maybe involved in multimerization.

However, the formation of multimers through the hinge domain cannot be explained for (iv) V_H_-Cµ1-Cµ2-Cγ3, which lacked the hinge domain but reacted strongly with the skin-related antigen. (iv) V_H_-Cµ1-Cµ2-Cγ3 formed more multimers than (vi) V_H_-Cµ1-Cµ2-Hinge-Cγ2-Cγ3 (Fig. 4). It has been demonstrated that the structure of the heavy chain portion of IgG can affect the binding activity of the antibodies to their antigens^21–23^. For example, the last 18 amino acids of IgM has been shown to mediate IgM polymerization^16, 17, 24^. Thus, placement of Ig domains in the molecules may impact the level and mode of multimerization, which may affect the overall structure and function of the chimeric molecule. Multimerization of antibodies is often caused by disulfide covalent bonds. A previous study showed that cysteine residues in the Cµ2 domain are involved in interchain disulfide bridges of mouse IgM^25^. However, our chimeric molecules may not form disulfide covalent associations. Non-reducing Western blotting analysis of gel filtration fractions detected monomeric forms even in higher molecular weight fractions (Fig. 4c), suggesting that the molecules formed non-covalent interactions. At present, the mechanism of multimer formation is unclear, but Ig domains responsible for multimer formation appear to be different between (vi) VH-Cµ1-Cµ2-Hinge-Cγ2-Cγ3 and (iv) VH-Cµ1-Cµ2-Cγ3.

To further improve multimerization, we introduced 2 previously described mutations into our molecules (Fig. 6). It was clearly shown that 309 C and µTP induced multimerization^16, 17^, but it was not demonstrated whether multimerization of IgG was required for functional antigen binding. The mutations greatly enhanced both multimerization and functionality of the hybrid molecules. Surprisingly and interestingly, even non-functional molecules became strongly functional after introducing the 2 mutations simultaneously. These results suggest that murine IgM can be converted into functional IgG-like molecules by mutating amino acid 309Q to 309 C and attaching the µTP to the C-terminus of the resultant molecules.

In a previous study, functional IgG form of anti-CD20 IgM antibody was made, after spatial conformations were determined with computer-guided homology modeling methods^18^. The functional antibody was in the single-chain Fv format^18^, but it was not clear whether the antibody formed multimers. In another study, a functional IgG form of anti-CD19 IgM antibody was made and expressed in SF9 insect cells^19^. Although the antibody was functional, it failed to be secreted from the cells, and it was also not clear whether the antibody formed multimers^19^. Thus, in this study, we succeeded in making IgG-like molecules from IgM, whose functionality is based on multimer formation. To the best of our knowledge, this is the first study demonstrating that multimerization is required for binding to the antigen. It is not clear whether this phenomenon is unique to our specific antibody or whether it is universal, but it must be worthy trying constructing multimers when an engineered IgG converted from IgM has no function.

(iv) V_H_-Cµ1-Cµ2-Cγ3 forms multimers readily (Fig. 4a) and reacts strongly with skin (Fig. 3d). However, it does not possess protein A/G binding activity (Fig. 2b), which is desirable in an IgG molecule for use in applications requiring specific IgG reagents. On the other hand, (vi) V_H_-Cµ1-Cµ2-Hinge-Cγ2-Cγ3 forms multimers (Fig. 4a), and is able to detect the antigen in a similar manner as (i) V_H_-Cµ1-Cµ2-Cµ3-Cµ4 and (iv) V_H_-Cµ1-Cµ2-Cγ3 (Fig. 3). Furthermore, it possesses all the properties of IgG molecules, including detection with specific IgG reagents and binding to protein A and G (Fig. 2b). Some of the functional multimers are smaller than the original IgM molecule and should be useful for studies requiring penetration of the antibodies into tissues thereby evading steric hindrance associated with large IgM molecule. Further promotion of multimerization by the double 309 C and µTP mutations have enabled us to identify the best IgG format of IgM molecules as (ii) V_H_-Cγ1-Hinge-Cγ2-Cγ3 with 309 C and µTP mutations, whose constant region consists of IgG domains only. Since this molecule significantly form multimers and strongly bind to Protein G, this antibody should be suitable for immunoprecipitation to identify the antigen.

In conclusion, we have successfully converted an IgM molecule into functional IgG-like molecules, which reacted strongly with a skin-related antigen. We demonstrated that the multimerization is important for functional binding of the chimeric antibodies to the antigen. The results in our study should provide important insights for future studies that aim to convert IgM to IgG-like antibody for research and therapeutic applications.

## Materials and Methods

### Ethics statement

The studies were approved by the internal review board of Kurume University School of medicine. All described experiments were performed following guidelines of ethical committee of Kurume University School of Medicine and in accordance with Declaration of Helsinki Principles. Informed consent was obtained from skin donors. Adjacent normal skin obtained during operation for other skin problems was used in this study. Unwanted human foreskin was also used.

### Isolation of RNA, RACE, cDNA synthesis and PCR

Hybridoma secreting an IgM antibody against a human skin antigen was established as described previously^4^. The cells were cultured in RPMI-1640 medium supplemented with 20% fetal calf serum (FCS). Total RNA was isolated with RNeasy Kit (Qiagen, Hilden, Germany) following the manufacturer’s instructions. cDNA synthesis and PCR was performed using the RNA-ligase mediated GeneRacer RACE Kit (Invitrogen, Carlsbad, CA) according to the manufacturer’s instructions. cDNA also was synthesized from total mouse spleen RNA (Biochain, Hayward, CA) with Superscript III reverse transcriptase (Invitrogen) and oligo (dT) primer. For PCR, forward primer included in the kit was used along with 5′-GGTTGAGCGCTAGCATGG-3′ and 5′-GGCGTCTCAGGACCTTTGTC-3′ reverse primers to amplify full-length mouse IgM heavy and light chains, respectively. PCR was performed with high fidelity PrimeStar Max DNA polymerase (Takara Bio, Shiga, Japan). The amplified products were cloned into PCR-blunt vector (Invitrogen) and sequenced to determine the variable regions of heavy and light chains.

### Construction of vectors encoding IgM and IgM/IgG molecules

Various primer pairs (Supplementary Table S1) were used to generate various portions of mouse IgM and IgG2a heavy chains by PCR. IgM forward primer, 5′-aaagcaaccggtgatatcACCATGGGATGGAGCTGG-3′ was used along with the listed IgM reverse primers to amplify IgM portions. IgG2a reverse primer, 5′-tacaagatctttgatatcGGTGTCTCTTTGGACCTGAGAGT-3′ was used along with the listed IgG forward primers to amplify IgG portions. For full-length IgM, 5′-tacaagatctttgatatcGGTTGAGCGCTAGCATGG reverse primer was used. For the kappa light chain, 5′-cggagtatacggatccAAAATGATGAGTCCTGCCCAG-3′ (forward) and 5′-taggcgtacgggatccGGCGTCTCAGGACCTTTGTC-3′ (reverse) primers were used. The primers were designed with additional 15 bases to the 5′-end (non-underlined lower case) to facilitate cloning by sequence- and ligation-independent cloning technology. Additional bases (underlined) were added to recreate BamHI or EcoRV restriction enzyme site. PCR was performed with PrimeStar Max DNA polymerase (Takara Bio). Amplified fragments were cloned simultaneously into pVITRO2-MCS dual expression vector (InvivoGen, San Diego, CA) that was digested with Fastdigest BamHI/EcoRV (Thermo Scientific, Hudson, NH), using Gibson assembly (New England BioLab, Beverly, MA) or In-fusion HD (Clontech, Pako Alto, CA) cloning kits. The heavy chain was inserted into BamHI site under control of mouse EF1α promoter and the light chain was inserted into EcoRV site under control of rat EF1α promoter. The hygromycin resistance gene in the original vector was replaced with puromycin resistance gene in some cases. The assembled fragments were transformed into NEBstable competent cells (New England BioLab) and transformants were selected on low salt LB agar plates supplemented with 25 µg/ml puromycin or 50 µg/ml hygromycin (InvivoGen) and 50 mM potassium phosphate buffer, pH 8.0. The sequences were verified by direct DNA sequencing.

### Mutagenesis to introduce 309 C and µTP mutations

Primers 5′-CCCATCtgtCACCAGGACTGGATGAGT-3′ (forward) and 5′-CTGGTGacaGATGGGGAGGGCACTGAC-3′ (reverse) were used to mutate the molecules to introduce 309 C mutation (lower case in primer). Whole circular plasmid was amplified as above. For introduction of µTP, primers 5′-atgtctgacacaggcggcacctgctatTGAGCTCAGCACCCACAAAAC-3′ (forward) and 5′-gatcagggagacattgtacagtgtgggTTTACCCGGAGTCCGGGA-3′ (reverse) were used. Half each of the 54-bp µTP (lower case) was attached to the forward and reverse primers. Primers were phosphorylated and PCR was performed as above. Whole linear plasmid PCR product was self-ligated using Ligation High kit (Toyobo). Plasmid was obtained after transformation and selection as described above. Sequences were confirmed by direct DNA sequencing.

### Establishment of stable cells and production of recombinant antibody

Lenti-X 293 T cells (Clontech), a form of HEK293T cells, were cultured in DMEM supplemented with 10% FCS. For transfections, cells were seeded into 12-well plates and transfected with 2.5 µg of plasmid DNA using 0.75 µl of Xfect transfection reagent (Clontech). Stable cells were generated by selection with 0.4–0.8 µg/ml puromycin or 200 µg/ml hygromycin and resistant cells were expanded and cultured until 80% confluent. After rinsing with PBS, medium was switched to MAB serum-free medium supplemented with 1% KOSR (Gibco-BRL, Grand-Island, NY) and cultured for 6 days. Antibodies were purified from culture supernatant using protein A (GeneScript, Piscataway, NJ), Protein G (Merck Millipore, Billerica, MA) or protein L (GeneScript) after buffer was exchanged to 20 mM sodium phosphate buffer, pH 7.2, containing 150 mM NaCl using Amicon centrifugation units. Antibodies were eluted with 100 mM glycine, pH 2.7, and immediately neutralized with 22 µl of 2 M Tris solution, per 1 ml of eluate.

### SDS-PAGE and Western blotting of mouse IgG and IgM

Samples were mixed with 5X SDS sample buffer with or without 5% β-mercaptoethanol, heated for 5 minutes at 80 °C (non-reducing) or 95 °C (reducing) and, separated by 5–20% gradient PAGE gel (ATTO, Nagoya, Japan). Proteins were transferred onto nitrocellulose membrane using iBlot 2 transfer device (Invitrogen). Membranes were blocked with 4% skim milk in TBS and incubated with HRP- or ALP- conjugated anti-mouse IgG (H + L or L) or anti-mouse IgM. After washing, proteins bands were visualized with TMB for HRP (ATTO) or 1-Step NBT/BCIP for ALP (Pierce Thermo Scientific, Hudson, NH).

### Protein G binding assay

Serum-free cell culture supernatant was diluted 5X in 20 mM sodium phosphate buffer, pH 7.2, containing 150 mM NaCl and added to washed protein G beads (Merck Millipore). The suspension was rotated at room temperature (RT) for 30 minutes. After washing extensively, bound material was eluted by heating at 80 °C in non-reducing SDS sample buffer, separated by SDS-PAGE and detected by immunoblotting as described above.

### Immunofluorescence of human skin sections

Normal human skin sections were blocked with 1% BSA/PBS for 30 min and incubated with antibodies at RT for 1 hour. After washing with PBS, sections were incubated with Alexa Fluor 488-conjugated anti-mouse IgG (H + L) (Invitrogen) diluted 1:1000 with 1% BSA/PBS. The sections were observed with BX51 fluorescence microscope (Olympus, Tokyo, Japan).

### Western blotting of human epidermal extract

Human skin, obtained at circumcision, was processed as described previously^26, 27^. Epidermis was homogenized in 20 mM Tris, pH 7.2, containing 150 mM NaCl and 1% Triton X-100. After centrifugation to remove insoluble materials, 20 μg total protein in the supernatant was used per lane in Western blotting. Blotted membranes were incubated with recombinant antibodies at RT for 2 hours. After washing, membranes were incubated with ALP-conjugated anti-mouse IgG (H + L), washed and detected with 1-Step NBT/BCIP.

### ELISA for IgM/IgG hybrid analysis

We developed an ELISA system to monitor the amount of recombinant antibodies in various samples. Maxisorp plates were coated with 100 μl of 0.5 μg/ml anti-mouse kappa light chain (Sigma-Aldrich, St. Louis, MO). Plates were blocked at RT for 3 hours with TBS containing 10% adult bovine serum (Sigma), 5% sucrose and 0.05% sodium azide, dried at RT for 2 hours, stored at 4 °C and used with 1 month. Antibody samples were diluted in TBS containing 2% adult bovine serum and 0.01% Tween 20. Diluted samples were incubated with the plates at RT for 1 hour and washed 4 times with TBS containing 0.05% Tween 20. The plates were incubated with 1:4,000 dilution of HRP-conjugated anti-mouse IgM (Santa Cruz Biotechnology, Santa Cruz, CA) or 1: 20,000 anti-mouse IgG (Nacalai Tesque, Kyoto, Japan) at RT for 1 hour. After washing, color was developed with TMB. Reactions were stopped with 0.5 M HCl and the optical density was read with plate reader at 450 nm. Antibody amounts were estimated with the built-in software of the plate reader using appropriate Ig as standard ranging from 3.125 to 200 ng/ml.

### Gel filtration analysis and immunofluorescent of normal human skin

Gel filtration analysis was carried out using Superdex 200 Increase 10/300 GL column (GE Healthcare) in PBS (pH 7.4) at 0.5 ml/min. 100 μl of purified antibodies was injected and optical density at 280 nm was continuously monitored by an AKTApurifier system (GE Healthcare). Samples were fractionated manually to measure the concentrations by ELISA and the antigen binding activity by immunofluorescence.

### Data Availability

All data generated or analyzed during this study are included in this published article and its Supplementary Information files.

## Electronic supplementary material

Supplementary Information
