# Supplementary material for: Multimerization is required for antigen binding activity of an engineered IgM/IgG chimeric antibody recognizing a skin-related antigen
Source: Sci Rep. 2017 Aug 15;7:8212. doi: 10.1038/s41598-017-08294-2 (PMC5557880; doi:10.1038/s41598-017-08294-2)
Supplement: Supplementary file 1 — Supplementary Information [file 41598_2017_8294_MOESM1_ESM.pdf]

Supplementary Information for

Multimerization is required for antigen binding activity of an engineered IgM/IgG chimeric antibody recognizing a skin-related antigen

Kwesi Teye[1], Koji Hashimoto[2], Sanae Numata[3], Kunihiro Ohta[2], Marek Haftek[4], Takashi Hashimoto[1]

[1] Kurume University Institute of Cutaneous Cell Biology, Kurume, Fukuoka, Japan
[2] Department of Life Sciences, Graduate School of Arts and Sciences, The University of Tokyo, Tokyo, Japan
[3] Division of Innovation and Education, Iwate Tohoku Medical Megabank Organization, Disaster Reconstruction Center, Iwate Medical University, Iwate, Japan
[4] University of Lyon 1, EA 4169 and CNRS, Lyon, France

Corresponding author:
Takashi Hashimoto. E-mail: hashimot@med.kurume-u.ac.jp or hashyt@gmail.com

This file include:

1. Supplementary Fig. S1
2. Supplementary Fig. S2
3. Supplementary Fig. S3
4. Supplementary Fig. S4
5. Supplementary Table S1

Supplementary Fig. S1

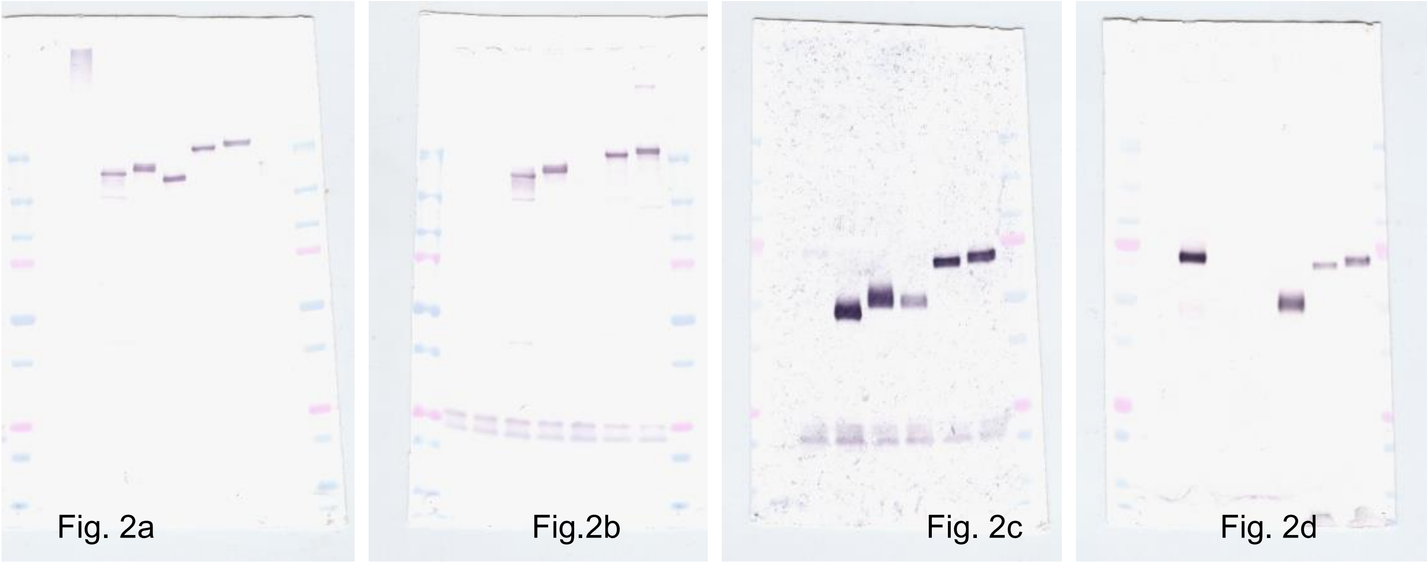

Supplementary Fig. S1. Full-length blots for the indicated figures in the article

Supplementary Fig. S2

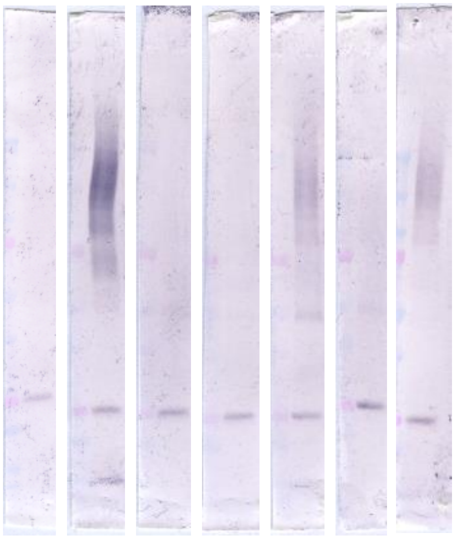

Fig. 3j

Supplementary Fig. S2. Full-length blots for the indicated figures in the article

Supplementary Fig. S3

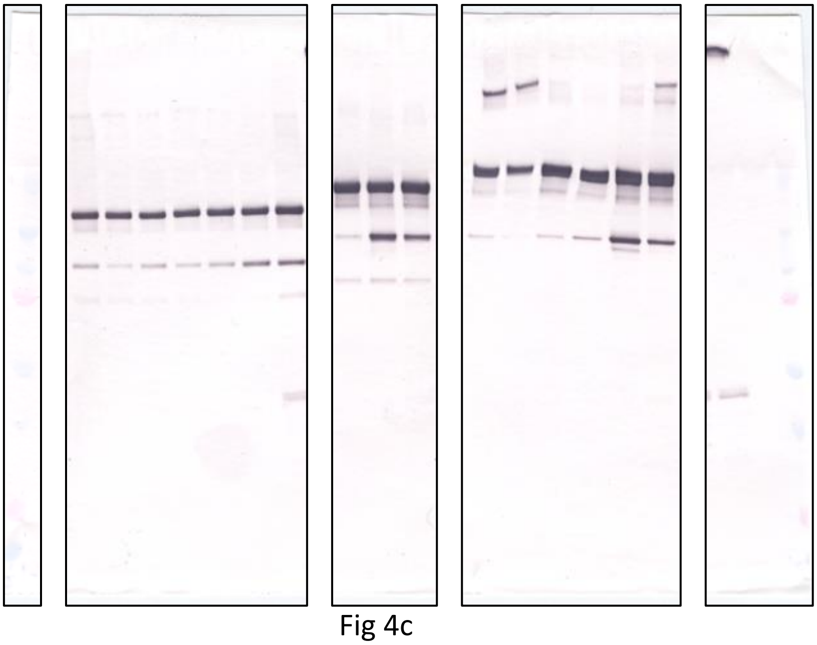

Supplementary Fig. S3. Full-length blots for the indicated figure in the article

Supplementary Fig. S4

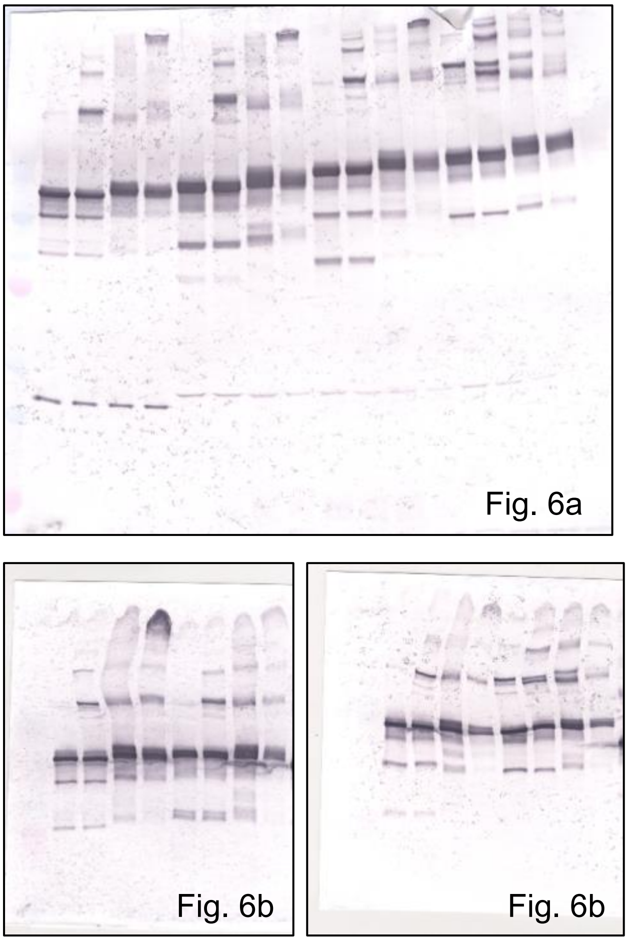

Supplementary Fig. S4. Full-length blots for the indicated figures in the article

Table S1. List of primers used for PCR amplifications

Molecule	IgM reverse primer	IgG forward primer
(ii) V_H-Cγ1-Hinge-Cγ2-Cγ3	ggctgttgttttggcTGCAGAGACAGTGACCAGAGTC	gtcactgtctctgcaGCCAAAACAACAGCCCCATC
(iii) V_H-Cμ1-Hinge-Cγ2-Cγ3	gggccctctgggctcTGGAATGGGCACATGCAG	catgtgcccattccaGAGCCCAGAGGGCCCACAAT
(iv) V_H-Cμ1-Cμ2-Cγ3	agctcttactgacccACTGGCAGCACATGTGGA	acatgtgctgccagtGGGTCAGTAAGAGCTCCACAGG
(v) V_H-Cμ1-Cμ2-Cγ2-Cγ3	caagaggttaggtgcACTGGCAGCACATGTGGAGG	acatgtgctgccagtGCACCTAACCTCTTGGGTGGAC
(vi) V_H-Cμ1-Cμ2-Hinge-Cγ2-Cγ3	gggccctctgggctcACTGGCAGCACATGTGGAGG	acatgtgctgccagtGAGCCCAGAGGGCCCACA
